# Assessing Emotional Distress in Adolescents: Psychometrics of the Spanish Version of the Social Emotional Distress Scale-Secondary

**DOI:** 10.1007/s10566-023-09758-5

**Published:** 2023-06-03

**Authors:** Tíscar Rodríguez-Jiménez, Verónica Vidal-Arenas, Raquel Falcó, Beatriz Moreno-Amador, Juan C. Marzo, José A. Piqueras

**Affiliations:** 1 Department of Psychology and Sociology, University of Zaragoza, Teruel, Spain; 2 Department of Basic and Clinical Psychology and Psychobiology, Jaume I University, Castellón de la Plana, Spain; 3 Department of Health Psychology, Miguel Hernández University of Elche, Alicante, Spain

**Keywords:** Social Emotional Distress Survey–Secondary, SEDS-S, Emotional distress, Adolescents, Screening, Mental health

## Abstract

**Background:**

The Social Emotional Distress Scale-Secondary (SEDS-S) is a short measure designed for comprehensive school-based mental health screening, particularly for using very brief self-reported measures of well-being and distress. Whereas prior studies have shown validity and reliability evidence for the English version, there is a lack of literature about its psychometric properties for Spanish-speaking youths.

**Objective:**

To examine the psychometric properties of the SEDS-S in a large sample of Spanish adolescents, providing evidence of its reliability, structure, convergent and discriminant validity, longitudinal and gender measurement invariance, and normative data.

**Methods:**

Participants were 5550 adolescents aged 12–18 years old. Test–retest reliability was examined using Cronbach’s alpha and McDonald’s omega coefficients, and evidence for convergent and discriminant validity was measured using Pearson’s correlation. Confirmatory factor analysis (CFA) was used to examine structure validity, while multigroup and longitudinal measurement invariance analysis was conducted for longitudinal and gender latent structure stability.

**Results:**

The CFA supported a unidimensional latent structure, which was also observed to be invariant between gender groups and over time. The scale showed evidence of reliability, with coefficients above .85. In addition, the SEDS-S score was positively related to measures assessing distress and negatively related to measures assessing well-being, thereby providing convergent/discriminant validity of the total scores.

**Conclusion:**

This study provides the first evidence of the reliability and validity of the Spanish version of the SEDS-S for assessing emotional distress among adolescents, cross-sectionally and longitudinally. Furthermore, findings indicated that SEDS-S could be a suitable assessment tool for screening and program evaluation purposes at different contexts beyond the school setting.

## Introduction

### Mental Health Models

Initially, mental health was conceptualized considering a taxonometric model characterized exclusively by the presence or absence of psychopathology. More recently, a comprehensive or two-dimensional mental health model was proposed as an alternative to this reductionist model, an approach that simultaneously considers indicators of psychological distress and socioemotional strengths (Furlong et al., 2014a; Keyes, 2005; Suldo & Shaffer, 2008). Internalizing symptoms and the underlying distress refer to psychological problems experienced inwardly, primarily characterized by negative affect such as anxiety, depression, and stress. These symptoms and distress are often concealed from others and may not be immediately visible to people outside the individual experiencing them (Piqueras et al., 2021a). The comprehensive mental health framework produces a complete picture of students’ psychosocial functioning, leading schools to strive to alleviate identified youths’ psychological distress. Therefore, measures of distress and strengths are necessary (Dowdy et al., 2018).

### Adolescence and Mental Health

Adolescence is a stage in which many physical, cognitive, and psychosocial changes occur that overlap with the acquisition of new roles and responsibilities (Steinberg, 2017). These changes can lead to detrimental outcomes among adolescents, such as feeling emotionally overwhelmed. Some authors explain this decline in mental health indicators as being due to the onset of adolescence with an increase in risky situations, which implies greater emotional, psychological, and social vulnerability (Solmi et al., 2021).

The incidence of psychosocial problems in young people is concerning, as emotional and behavioral disorders are associated with short- and long-term effects, such as suicide attempts, substance use, impaired social functioning, and high dropout rates (Erskine et al., 2016; Fonseca-Pedrero et al., 2020; Kern et al., 2013). In Spain, data from the latest Spanish National Health Survey in 2017, which collected parent-based information on 6,106 minors aged 0–14 years, have indicated that 1% of children have a mental health problem, obtaining a prevalence of depression and anxiety of 0.6%, with a higher frequency in girls than in boys (Ministerio de Sanidad, Consumo y Bienestar, 2017). Additionally, Canals et al. (2019) found that anxiety disorders in early adolescence were an important public health problem with a mean prevalence of 11.8%, with differences in favor of girls. Recently, according to the Multidisciplinary Working Group on Child and Adolescent Mental Health from the Spanish Association of Primary Care Pediatrics, the pandemic has led to an increase of up to 47% in mental health disorders in minors (Asociación Española de Pediatría de Atención Primaria, 2022). Specifically, this statement warns that cases of anxiety and depression and Attention Deficit Hyperactivity Disorder diagnoses have been multiplied by three to four since 2019, and suicidal behaviors have increased by up to 59%. This report highlights the increase of psychological and behavioral changes, especially emotional symptoms, among Spanish children and adolescents during the COVID-19 pandemic. Specifically, the data show that the prevalence of anxious and depressive symptoms were higher than reported before the pandemic; specifically, 19% of children and adolescents showed depressive symptoms, and 38% presented anxiety symptomatology (Francisco et al., 2020; Orgilés et al., 2021). Despite advances in the assessment of mental health problems, most of these youths are unidentified and unattended (Catalano & Kellogg, 2020).

### Universal School-Based Screen

Universal school-based screening of academic performance and mental health is the first step in identifying students needing support. Universal mental health screening requires that all students’ current functioning be screened to determine possible significant symptoms of distress. This approach may be especially beneficial in identifying students with internalizing conditions, such as depression, anxiety, or suicidal ideation because they are not as easily identified compared to their peers with externalizing conditions, such as physical aggression, verbal bullying, relational aggression, defiance, theft, or vandalism (Weist et al., 2007). Deficit measures only identify 15—20% of students with substantial psychosocial problems, such as internalizing and externalizing problems. Given the importance of universal screening processes in creating comprehensive mental health programs in schools, it is critical to validate measures that provide information about students’ mental health (Furlong et al., 2020).

Various reasons justify the need for valid and reliable screening instruments for health problems in adolescents (Tran et al., 2019). One is that it is necessary and helpful for clinical practice to assess both emotional disorders and symptoms and related conditions in children and adolescents when making a first diagnostic approach, and also to screen for these symptoms in the general population (Ebesutani et al., 2012). According to these authors, another reason is that any approach to assessing these disorders involves the lack of time for the mental health professional to perform a diagnostic assessment, the most common practice being self-report tests to detect these symptoms. Moreover, self-report instruments have proven to be the first choice for screening and detecting anxiety and depression, with undoubted advantages over other techniques such as clinical interviews or observational techniques (Chorpita & Southam-Gerow, 2007).

### Alternative Approaches to Traditional Classifications for Mental Health

Over the past 20 years, there have been alternative proposals to traditional categorical classifications for mental health. These alternatives could be considered evidence-based quantitative organizations of psychopathology (e.g., Achenbach & Rescorla, 2007; Clark & Watson, 2006; Kessler et al., 2002; Kotov et al., 2017; Krueger, 1999; Watson, 2005). These quantitative nosologies, rather than being constructed top-down, have emerged from the independent work of multiple research groups attempting to understand the natural organization of psychopathology. The most recent example is the Hierarchical Taxonomy of Psychopathology initiative (Kotov et al., 2021). Accordingly, internalizing disorders can be differentiated into two sets: distress or distress disorders such as Generalized Anxiety Disorder (GAD), Post-Traumatic Stress Disorder (PTSD), Major Depressive Disorder (MDD), and Dysthymic Disorder (DD); and fear/anxiety disorders (such as panic and phobias) (Clark & Watson, 2006; Krueger, 1999; Watson, 2005). From this framework, expressions such as “depression-anxiety disorders spectrum,” “emotional disorders spectrum,” “emotional disorders continuum,” or “internalizing spectrum” have been used. They include different nosological entities such as distress and fear disorders, emphasizing that all these anxiety- and depression-related disorders share an “internalizing factor” (Watson, 2005). For example, the most recent and promising model, the HiTOP model, considers that there is a super-spectrum—a general psychopathology factor—that comprises some spectra, such as internalizing, somatoform, externalizing (disinhibited and antagonistic), thought disorders, and detachment-related spectra. The internalizing spectrum would also include subfactors such as sexual problems, eating pathology, fear, distress, and, partially, mania. The fear subfactor would include some syndromes/disorders, such as social phobia, agoraphobia, specific phobia, separation anxiety disorder, panic disorder, and obsessive–compulsive disorder. In contrast, the distress subfactor would include MDD, DD, GAD, PTSD, and borderline personality disorder. All these syndromes/disorders arise from symptomatic components and maladaptive traits (Components) and symptoms (Signs and Symptoms) (for more information, see Fig. 2. *Spectra of the Hierarchical Taxonomy of Psychopathology* in Kotov et al., 2017).

A particularly useful and novel integrative model for community school settings has been the so-called Bidimensional Mental Health Model (BMHM; Geenspoon & Safklofske, 2001) and later the Dual-Factor Model (DFM; Suldo & Shaffer, 2008) which suggests the use of mental health tools that include the two facets of mental health, positive indicators of wellness-health (i.e., subjective well-being) and traditional negative indicators of distress-illness (i.e., psychopathology) to comprehensively measure mental health. Consisting with a BMHM or DFM approach, universal school-based mental health screening measures simultaneously should assess symptoms of wellness and distress (Furlong et al., 2022). This perspective emphasizes that mental health encompasses a balance of wellness-health and distress-illness in at school setting. It is this universal screening framework that recognizes the need to develop and validate brief, unidimensional distress measures complementing the measurement of positive indicators of mental health or well-being instead of comprehensive symptom measures, which are inefficient and impractical for universal screening applications due to cost and the high number of items (Furlong et al., 2022; Rivera-Riquelme et al., 2019).

### Measures of Global Psychological Distress for Children and Adolescents

Following these proposals, dimensional measures of global psychological distress have emerged, which are important for distinguishing community cases based on severity rather than purely on a diagnosis. Furthermore, according to Cuijpers et al. (2009) or Spence and Rapee (2022), adolescents are more easily screened if the procedure is brief, quick, and easy to read, so it is crucial to have concise measures that are reliable and valid in identifying mental health problems in young people, as they play a vital role in facilitating early intervention. Furthermore, while detailed assessment measures are necessary to accurately identify mental health problems, target intervention content and evaluate outcomes, they can be time-consuming for both participants and professionals and require considerable resources, so the use of such detailed measures may be limited in large-scale community screening and population-level research with youth. Therefore, there is a need for brief, standardized, and psychometrically sound instruments that are highly sensitive in indicating the possible presence of mental health disorders, such as global psychological distress, while maintaining good specificity to avoid false identification of large numbers of young people without clinical levels of the problem in question. Some of the measures that meet these requirements are the Kessler Psychological Distress Scale (K10; Kessler et al., 2002), or the Social Emotional Distress Scale-Secondary (SEDS-S; Dowdy et al., 2018).

Both tools, SEDS-S and K10, share many common features. They have the same time frame, covering the last month and last 30 days, and similar response options ranging from 1 (Not at all true) to 4 (Very true) for SEDS-S, and from 1 (Not at all) to 5 (Every time) for K10. Additionally, both questionnaires have similar item content. Thus, SEDS-S focuses on anxiety and depression, with approximately half of its items related to anxiety (such as difficulty breathing, feeling ashamed of oneself, being tense and nervous, difficulty relaxing, etc.) and the rest on depression (such as feeling sad and down, difficulty getting excited, etc.) (Dowdy et al., 2018). On the other hand, K10 includes two sets of items: anxiety, which includes items like Fatigued, Nervous, Very nervous, Restless, Very restless, and No energy; and depression, which includes items like Hopeless, Sad, Very sad, and Useless (Larzabal-Fernandez et al., 2023).

However, they also have differential characteristics, which made us opt for the SEDS-S. The K10 was not specifically designed for children and adolescents, but K10 and its 6-item version (K6) have been used with adolescents in various studies and demonstrated quite good psychometric properties in diverse settings including China, Indonesia, USA, and Ecuador (Chan & Fung, 2014; Larzabal-Fernandez et al., 2023; Mewton et al., 2016; Tran et al., 2019). However, one of the most recent studies (Larzabal-Fernandez et al., 2023), points out that the evidence of validity for K10 in the adolescent population is quite limited and controversial and is reduced to two studies, both using the 6-item version (K6) (Mewton et al., 2016; Tran et al., 2019) and that they had not been able to find studies investigating the psychometric properties of the K10 in adolescents. Furthermore, in the study with Ecuadorian adolescents, they concluded that the 9-item version of the K10 was the most appropriate for this population. Overall, the K10 appears to be a reliable and valid measure of psychological distress among adolescents, but it was not specifically designed for adolescents, so, as with any measure, it is important to consider the specific context and population being studied to determine the most appropriate measure to use.

The SEDS-S (Dowdy et al., 2018), on the other hand, was specifically developed for use with secondary school students and has demonstrated good psychometric properties in this population. It assesses emotional and social distress, which may be particularly relevant for adolescents who are navigating a range of social and emotional challenges during this developmental period.

Therefore, both scales can be used with adolescents and have been shown to be effective in assessing psychological distress among adolescents. However, the choice of which one to use will depend on the specific research question and the nature of the population being studied.

### The Current Study

A question arises from the previous paragraph: why was the SEDS-S (Dowdy et al., 2018) selected instead of adapting the K10 (Kessler et al., 2002) or any other brief measure of internalizing distress? From our point of view, it should be mentioned that the SEDS-S made it a better fit for our study population and research question. Thus, the SEDS-S was designed specifically for adolescents and for comprehensive school-based mental health screening (Dowdy et al., 2018, 2022) and was therefore particularly well-suited for our study of Spanish-speaking adolescents. Alternatively, the SEDS-S has been shown to have similar psychometric properties or validity evidence to other similar measures, such as K-10 making it an equivalent reliable and valid measure of emotional distress in this population.

The SEDS-S is an assessment instrument that aims to assess youths’ emotional distress in the school context, but it does not measure syndrome patterns (Dowdy et al., 2018). This emphasis on non-pathological emotional distress lends itself to broad-based surveillance screening, which aligns well with universal school-based screening approaches. The original version consists of 10 items with four response options ranging from 1(*not true*) to 4 (*absolutely true*). It has good psychometric properties with high reliability and good model fit for unidimensional structure (Dowdy et al., 2018). Furthermore, Furlong et al. (2021) supported the unidimensional factor structure of the SEDS-S, and other studies have also reported good internal consistency indices, with Cronbach’s alpha or omega between 0.91 and 0.95 (Chan et al., 2022a, 2022b; Furlong et al., 2020, 2021; Kim et al., 2019; Maupin, 2021). More recently, a study examined the psychometrics properties of the 5-item short version of SEDS (SEDS-S-Brief; Dowdy et al., 2022). Overall, this study found evidence to support a unidimensional latent structure, which has also shown invariant across time, sex, grade level, thereby supporting its use across diverse groups in schools. Finally, Furlong et al. (2022) used the named “25–25-50 cut-score approach for the measure of distress factor” as a screening tool in a BDMH/DFM-based approach.

However, despite these promising findings, as far we know, there is no evidence about the psychometrics properties of the Spanish versions of SEDS-S. Therfore, the present study aims to examine the psychometric properties of the SEDS-S in a large sample of Spanish adolescents, providing evidence of reliability and factorial structure, convergent and discriminant validity, longitudinal and gender invariance, and normative data. According to our first hypothesis, we expected to find evidence for the unidimensionality of the SEDS-S. Our second hypothesis postulated that the SEDS-S would demonstrate measurement invariance across gender groups and over time and that female participants would score higher on general psychological distress compared to male participants. The third hypothesis was that the SEDS-S would show evidence of convergent and criterion validity, as indicated by significant relationships between SEDS-S scores and well-established measures of distress (i.e., Mental Health Continuum-Short Form, Youth-Pediatric Symptom Checklist-17) and well-being (i.e., Social-Emotional Health Survey-Secondary, KIDSCREEN-10 Index). Our fourth hypothesis stated that the estimated reliability of the SEDS-S scores would exceed a threshold of 0.70. Finally, we expected that the normative data obtained from the SEDS-S would be consistent with the only previous study that has included percentiles for US adolescents (Furlong et al., 2022), so this would support the SEDS-S is valuable for screening purposes.

## Method

### Participants and Procedure

This study presents an empirical design as follows a quantitative, observational, and multicentric methodology (Montero & León, 2007). Adolescents aged from 12 to 18 participated in a longitudinal project. Two waves of data were collected with a six-month interval (T1, *n* = 5,550 adolescents, *M*_age_ = 14.17 years [*SD* = 1.51], 50.8% female; T2, *n* = 2,168, *M*_age_ = 13.96 years [*SD* = 1.39], 49.9% female). The participants were enrolled in Spanish secondary education grades equivalent to USA middle and high school from 7th (age 12–13) to 12th grade (age 17–18). The study was approved by the Universidad Miguel Hernández (UMH) Project Evaluation Committee with reference number “DPS.JPR.02.17”. Once the project was approved, a quota sampling was carried out in two areas of south-eastern Spain: the province of Alicante, belonging to the Valencian Community and the Autonomous Community of Region of Murcia, making a random selection of secondary schools based on ownership (public/non-public schools) and regional geographical areas (9 areas in Alicante and 21 in Murcia). After 100 schools were contacted, 13 from Alicante and 21 from Murcia agreed to participate, with 34 secondary schools (65.2/34.8% public/non-public and 87/13% secular/Catholic schools of the total number of schools). After the schools had accepted, we requested written informed consent from the adolescent participants and their parents/legal guardians to participate in the research. The data collection for both assessment waves (T1 and T2) was carried out in the schools and supervised by the research staff. The self-report assessment protocol was applied individually through the online survey tool LimeSurvey ©. Participation was voluntary, and the adolescents did not receive any incentive for their collaboration. In contrast, each school received a feedback report by the class group of the results on bidimensional mental health.

### Measures

#### Social-Emotional Distress Survey-Secondary (SEDS-S; Dowdy et al., 2018)

The Social-Emotional Distress Survey-Secondary (SEDS-S) is a 10-item behavioral screening questionnaire designed to measure internalizing distress. In their study, Dowdy et al. (2018) found significant positive relations between the SEDS-S distress factor and symptoms of anxiety and depression and a significant negative association with life satisfaction and strengths scores.

#### Mental Health Continuum-Short Form (MHC-SF; Keyes et al., 2008; Piqueras et al., 2022)

This measure provides self-reported well-being, divided into 3 sub-factors: Psychological (6 items) (PWB), Emotional (3 items) (EWB), and Social well-being (5 items) (SWB). Each item has six response options on the frequency of subjective well-being symptoms in the last month, ranging from 1 (*never*) to 6 (*always*). The MHC-SF has received support in adolescent population from many international studies, including a recent study with a large sample of Spanish adolescents, which showed reliability and validity, with the bi-factor model being invariant over time and across gender groups (Piqueras et al., 2022).

#### Youth-Pediatric Symptom Checklist-17 (PSC-17-Y; Jellinek et al., 1999; Piqueras et al., 2021b)

The Spanish version of PSC-17-Y was administrated to assess psychosocial problems among youths. Specifically, through 17 items with three response options (0 = *never*, 1 = *sometimes*, 2 = *often*), the PSC-17-Y assesses three types of psychopathology problems: internalizing (i.e., depression and anxiety), externalizing (i.e., disruptive behavior), and attention deficit hyperactivity (ADH). The PSC-17-Y has received support for pediatric practice in three separate works (Bergmann et al., 2020; Gardner et al., 1999; Parker et al., 2019), and validity and reliability evidence among Spanish youths have also been reported (Piqueras et al., 2021b).

#### KIDSCREEN-10 Index (Ravens-Sieberer et al., 2010)

This is a 10-item unidimensional scale measuring health-related quality of life (HRQoL) in healthy and chronically ill children and adolescents. For each item, five response options are provided: “*not at all”, “slightly”, “moderately”, “very much”,* and *“extremely*”. It was developed to identify children at risk in terms of subjective health and suggests appropriate early interventions. The instrument provides an overall HRQoL index covering the physical, psychological, and social facets of HRQoL. Reliability indices (Cronbach’s alpha) reach 0.82, and test–retest reliability within two weeks reaches 0.55 (Ravens-Sieberer et al., 2010).

#### Social-Emotional Health Survey-Secondary (SEHS-S; Furlong et al., 2014b)

To measure the level of socio-emotional competence through the components of the latent construct Covitality among youth, the 36-item form of the Spanish version of the Social-Emotional Health Survey–Secondary was used (SEHS–S; Piqueras et al., 2019). The students’ responses are recorded on a four-point scale ranging from 1 (*not at all true of me*) to 4 (*very true of me*).

### Data Analyses

All analyses were conducted with SPSS v.25 and Mplus 8.4. First, we examined item distribution and frequencies of the items. Previously, the analysis of outliers was carried out by graphically representing the results (box diagrams). Although outliers were detected, we decided not to remove them from the sample for ecological validity. Next, confirmatory factor analyses (CFA) were conducted to test the unidimensionality of the study scale. We used maximum likelihood estimation (ML). Finally, we tested the model’s goodness of fit using the comparative fit index (CFI), the Tucker–Lewis index (TLI) and the root mean square error of approximation (RMSEA). For CFI and TLI, a value ≥ 0.90 indicates an acceptable fit, and a value ≥ 0.95, indicates an optimal fit. RMSEA values ≤ 0.06 indicate optimal fit (Marsh et al., 2004). Subsequently, we tested the SEDS-S’s measurement invariance across gender groups and over time (i.e., multi-group and longitudinal measurement invariance; Byrne & Watkins, 2003). In particular, three levels of measurement invariance were tested: (1) *configural* (testing whether all items load on the proposed factor), (2) *metric* (testing whether item-factor loadings are similar across groups), and (3) *scalar* (testing whether the unstandardized item thresholds are similar across groups). To indicate a significant decrement in fit when testing for measurement invariance, we used model comparison criteria of ΔCFI and ΔTFI ≥ 0.01 (i.e., a decrease indicates poorer fit; Cheung & Rensvold, 2002) and ΔRMSEA ≥ 0.015 (i.e., an increase indicates poorer fit; Chen, 2007). When there is scalar measurement invariance, the comparison of factor means across groups is allowed (Dimitrov, 2012). Consequently, we calculated gender differences. We also estimated Cohen’s *d* index (standardized mean difference), which allows for evaluating the effect size of the obtained differences (Cohen, 1988).

Convergent and criterion validity were evaluated by calculating the Pearson correlation coefficients between SEDS-S scores and different well-established measures of distress (i.e., MHC-SF, PSC-17-Y) and well-being (i.e., SEHS-S, KIDSCREEN-10 Index). Cohen’s criteria were used to estimate the magnitude of the associations (Cohen, 1988). Finally, we also calculated Cronbach’s alpha (Cronbach, 1951) and ordinal omega coefficients (McDonald, 1999) to test the reliability of the scores. The normative data for the SEDS-S are presented as centiles (75th and 90th).

## Results

### Structure Validity

Adequate fit indices for the baseline model were observed (Table 1). Results for multi-group measurement invariance across gender groups and longitudinal measurement invariance analysis are summarized in Table 1. When we tested the configural invariance across gender groups and waves, we found acceptable to optimal fit indices. Metric and scalar invariance were also found across study groups and time, as changes in CFI and TLI, and RMSEA were lower/higher than 0.010 and 0.015, respectively (Table 1).

**Table 1:** Goodness-of-fit for the unidimensional structure of SEDS-S

	Overall Fit Indices					Comparative Fit Indices			
	χ^2^	*df*	CFI	TLI	RMSEA [90% CI]	Model comparison	ΔCFI	ΔTLI	ΔRMSEA
*Baseline*									
Unidimensional structure	1242.15	35	.945	.930	.078 [.075, .082]	–			
*Longitudinal measurement invariance*									
1. Configural	2199.729	169	.913	.903	.046 [.044, .047]				
2. Metric	2246.472	178	.912	.906	.045 [.043, .047]	1 versus 2	− .001	.003	− .001
3. Scalar	2427.904	188	.904	.903	.046 [.044, .047]	2 versus 3	− .008	− .003	.001
*Multigroup measurement invariance across gender*									
1. Configural	916.177	70	.942	.925	.066 [.062, .070]				
2. Metric	970.799	79	.939	.930	.064 [.060, .067]	1 versus 2	− .003	− .005	− .002
3. Scalar	1062.286	88	.933	.932	.063 [.060, .066]	2 versus 3	− .006	.002	− .001

### Reliability Coefficients and Descriptive Analyses

The Cronbach’s alphas and omega were adequate (see Table 2) in the whole sample between gender groups and across time, with internal coefficients above 0.85. Moreover, means comparisons of SEDS-S scores showed significant differences between gender groups (higher scores in females than males). The means for the items ranged between 1.39 (Item 1) and 2.16 (Item 2), and their standard deviation ranged between 0.76 (Item 10) and 1.08 (Item 2). Factor loadings were all significant (*p* < 0.001) and salient (i.e., equal to or higher than 0.486; see Table 3). Finally, most items had skewness and kurtosis values within the ± 2 range, but Items 1 and 10 presented kurtosis higher than 3, confirming that they were not normally distributed (see Table 3).

**Table 2:** Descriptive statistics across study group﻿s

	Cronbach’s Alpha (95% CI)		Omega (95% CI)		Cronbach’s Alpha (95% CI)		Omega (95% CI)		Mean score (*SD*)		*d*^*a*^
	Time 1	Time 2	Time 1	Time 2	Female	Male	Female	Male	Female	Male	*Male-Female*
SEDS-S	.88 (.88, .89)	.86 (.85, .86)	.89 (.88, .89)	.88 (.88, .89)	.89 (.43, .46)	.86 (.37, .40)	.89 (.89, .90)	.87 (.86, .87)	19.14 (6.95)	16.94 (5.97)	.34***
MHC-Emotional	.79 (.78, .80)	.86 (.58, .87)	.81 (.79, .82)	.81 (.79, .83)	.80 (.55, .59)	.77 (.51, .55)	.82 (.80, .83)	.79 (.77, .81)	13.83 (3.30)	14.31 (3.06)	−.15***
MHC-Social	.84 (.83, .84)	.86 (.85, .87)	.84 (.84, .85)	.84 (.83, .85)	.85 (.51, .54)	.83 (.47, .51)	.86 (.85, .86)	.83 (.82, .84)	19.66 (5.60)	20.32 (5.50)	−.12***
MHC-Psychological	.86 (.85, .87)	.89 (.88, .90)	.86 (.85, .86)	.859 (.85, .87)	.86 (.50, .53)	.86 (.48, .52)	.86 (.85, .87)	.86 (.85, .87)	28.02 (5.75)	28.48 (5.59)	−.08**
PSC-Y-Attention	.62 (.60, .63)	.64 (.61, .66)	.70 (.69, .72)	.71 (.69, .71)	.61 (.22, .26)	.63 (.24, .27)	.59 (.56, .60)	.60 (.58, .62)	4.45 (1.98)	4.46 (2.07)	−.00
PSC-Y-Internalizing	.75 (.74, .76)	.75 (.73, .76)	.75 (.74, .76)	.75 (.73, .77)	.76 (.37, .41)	.73 (.33, .36)	.76 (.75, .78)	.72 (.70, .74)	3.74 (2.37)	2.79 (2.17)	.42***
PSC-Y-Externalizing	.66 (.64, .67)	.64 (.62, .66)	.66 (.65, .68)	.66 (.63, .68)	.65 (.19, .22)	.67 (.43, .46)	.66 (.64, .68)	.67 (.65, .69)	2.54 (2.07)	2.80 (2.15)	−.12***
KIDSCREEN-10	.85 (.84, .85)	.84 (.82, .84)	.85 (.84, .85)	.85 (.84, .86)	.86 (.36, .38)	.83 (.21, .24)	.86 (.85, .87)	.83 (.82, .84)	37.38 (7.37)	39.80 (6.44)	−.35***
SEHS	.91 (.90, .91)	.93 (.93, .93)	.91 (.90, .91)	.90 (.89, .91)	.91 (.21, .23)	.90 (.20, .21)	.91 (.91, .92)	.90 (.90, .91)	110.60 (14.76)	112.19 (14.11)	−.11

^a^Student’s T-test o test gender differences

*p < .05

**p < .01

***p < .001

**Table 3:** Descriptive statistics of items

In the past month,… [Durante el último mes,…]	Mean (*SD*)	Skewness	Kurtosis	Factor Loadings
Item 1. I had a hard time breathing because I was anxious	1.39 (.790﻿)	2.11	3.59	.553^***^
[He tenido dificultad para respirar porque estaba ansiosa/o]				
Item 2. I worried that I would embarrass myself in front of others	2.16 (1.08)	.476	− 1.07	.486^***^
[Me ha preocupado hacer el ridículo delante de otras personas]				
Item 3. I was tense and uptight	2.15 (1.03)	.466	− .93	.696^***^
[He estado tensa/o]				
Item 4. I had a hard time relaxing	1.96 (.99)	.716	− .57	.748^***^
[He tenido dificultad para relajarme]				
Item 5. I felt sad and down	2.07 (1.04)	.593	− .84	.759^***^
[Me he sentido triste y desanimada/o]				
Item 6. I was easily irritated	2.05 (1.04)	.582	− .87	.678^***^
[Me he enfadado con mayor facilidad]				
Item 7. It was hard for me to cope and I thought I would panic	1.51 (.88)	1.64	1.59	.756^***^
[Me ha costado afrontar el día a día y he pensado que podría tener un ataque de nervios]				
Item 8. It was hard for me to get excited about anything	1.68 (.914)	1.19	.40	.622^***^
[Me ha costado ilusionarme por cualquier cosa]				
Item 9. I was easily annoyed and sensitive	1.69 (.89)	1.18	.46	.716^***^
[He estado más irritable/susceptible]				
Item 10. I was scared for no good reason	1.40 (.76)	1.98	3.22	.539^***^
[He estado asustada/o sin motivo]				

Likert-type response options used: English version: 1 Not at all true // 2 A little true // 3 Pretty much true // 4 Very much true [Spanish version: 1 Nada cierto // 2 Algo cierto // 3 Bastante cierto // 4 Totalmente cierto]

### Convergent/Discriminant Validity

Pearson correlations analyses showed significant (*p* < 0.001) and positive associations between SEDS-S scores and psychopathology subscales (internalizing, *r* = 0.670; externalizing, *r* = 0.356; attention, *r* = 0.351), and significant (*p* < 0.001) whereas negative associations with subjective well-being (emotional, *r* = -0.438; social, *r* = -0.379; psychological, *r* = -0.415), socio-emotional competences (*r* = -0.393), and quality of life (*r* = -0.602) were also observed.

### Normative Data

An ANOVA was performed including gender and age as fixed factors. Statistically significant differences were found for gender, *F*(1) = 93.875, *p* = 0.001, *η*^*2*^ = 0.17; age, *F*(6) = 34.635, *p* = 0.001, *η*^*2*^ = 0.036; and the Gender x Age interaction, *F*(6) = 7.483, *p* = 0.001, *η*^*2*^ = 0.008. In all cases, the effect sizes were small to medium (*η*^*2*^ = 0.010 –0.060). Centiles are presented for the total sample and by age and gender (see Table 4).

**Table 4:** Normative information about SEDS-S for adolescents (centile scores)

Centiles	General (*n* = 5550)	12		13		14		15		16		17		18	
		Girls (*n* = 392)	Boys (*n* = 365)	Girls (*n* = 689)	Boys (*n* = 671)	Girls (*n* = 654)	Boys (*n* = 588)	Girls (*n* = 537)	Boys (*n* = 523)	Girls (*n* = 362)	Boys ( *n* = 375)	Girls (*n* = 130)	Boys (*n* = 151)	Girls (*n* = 58)	Boys *n* = 55)
1	10.00	10.00	10.00	10.00	10.00	10.00	10.00	10.00	10.00	10.00	10.00	10.00	10.00	11.00	10.00
5	10.00	10.00	10.00	10.00	10.00	11.00	10.00	11.00	10.00	12.00	10.00	11.55	10.00	11.00	11.80
10	11.00	10.00	10.00	11.00	10.00	12.00	10.00	12.00	11.00	13.00	11.00	13.00	12.00	13.00	12.00
15	12.00	11.00	11.00	11.00	11.00	12.00	11.00	13.00	12.00	14.00	12.00	14.00	12.00	13.00	13.00
20	12.00	11.00	11.00	12.00	11.00	13.00	11.00	14.00	12.00	15.00	12.00	15.00	13.00	14.00	14.00
25	13.00	12.00	11.00	13.00	12.00	14.00	12.00	14.00	13.00	16.00	13.00	15.00	14.00	15.75	14.00
30	13.00	12.00	12.00	13.00	12.00	15.00	13.00	15.00	13.00	17.00	14.00	17.00	14.00	16.00	15.00
35	14.00	13.00	12.00	14.00	13.00	15.25	13.00	16.00	14.00	18.00	14.00	18.00	15.00	17.00	16.00
40	15.00	13.20	13.00	15.00	14.00	16.00	14.00	17.00	15.00	19.00	15.00	19.00	16.00	18.00	16.00
45	16.00	14.00	13.00	15.00	14.00	17.00	14.00	18.00	15.00	20.00	15.00	20.00	17.00	19.00	17.00
50	16.00	15.00	14.00	16.00	15.00	18.00	15.00	19.00	16.00	21.00	16.00	21.00	17.00	20.00	18.00
55	17.00	15.00	15.00	17.00	16.00	19.00	15.00	20.00	17.00	22.00	17.00	22.00	19.00	21.00	18.80
60	18.00	16.00	15.60	18.00	17.00	20.00	16.00	21.80	17.00	23.00	18.00	23.60	20.00	21.00	21.00
65	19.00	17.00	17.00	19.00	18.00	21.00	17.00	23.00	18.00	24.00	19.00	24.00	21.00	22.35	24.00
70	21.00	18.00	17.00	19.00	19.00	22.00	18.00	24.00	19.00	25.00	20.00	26.00	22.00	24.00	24.00
**75**	**22.00**	**19.00**	**18.00**	**21.00**	**20.00**	**23.00**	**20.00**	**26.00**	**20.00**	**27.00**	**21.00**	**27.00**	**23.00**	**25.00**	**24.00**
80	24.00	20.00	20.00	23.00	21.00	25.00	22.00	27.00	22.00	28.00	22.80	29.80	24.00	27.20	25.00
85	26.00	22.00	21.00	25.00	23.00	27.00	23.65	29.00	24.00	30.00	25.00	31.00	25.00	29.00	25.60
**90**	**28.00**	**25.70**	**24.00**	**27.00**	**25.00**	**30.00**	**26.00**	**30.00**	**25.60**	**32.00**	**27.00**	**32.90**	**27.00**	**31.30**	**26.40**
95	31.00	31.00	26.70	30.50	28.00	33.00	30.00	34.00	28.00	33.85	30.00	36.00	30.40	36.05	28.60
**99**	**37.00**	**39.00**	**37.34**	**36.10**	**34.28**	**37.00**	**34.11**	**38.00**	**34.00**	**36.37**	**34.24**	**38.00**	**35.96**		

## Discussion

Given the reported increase in emotional symptoms in children and adolescents during the COVID-19 pandemic (Orgilés et al., 2021), prevention or treatment programs for this population should be based on prevalence data. In addition, some studies indicate a need for brief validated instruments for universal school-based screening (Furlong et al., 2020). Therefore, this study aimed to determine the psychometric properties of the SEDS-S in a sample of Spanish adolescents. The analyses show that this instrument works adequately to assess psychological distress in a general population of adolescents aged 12 to 18 years.

The results of the CFA conducted in this study confirmed the unidimensional structure obtained in the original study (Dowdy et al., 2018) and in that of Furlong et al. (2021), both with English-speaking samples. In addition, our finding is also consistent with the Brief version of SEDS-S, with satisfactory unidimensional model fit (Dowdy et al., 2022).

On the other hand, to our knowledge, our study provided the first evidence of longitudinal invariance, indicating that SEDS total scores can be compared over time, allowing changes in scores to be assessed. These results imply that it can be concluded that growth or development in observed scores over time can be attributed to actual development or changes in the construct under investigation and not to measurement problems (Millsap & Cham, 2012). Furthermore, gender invariance analysis supported that psychological distress, as measured by the SEDS-S, was measured similarly in boys and girls, as found in a previous doctoral dissertation (Maupin, 2021). This finding was consistent with the SEDS-S-Brief, consisting of 5 items from the original 10-item SEDS-S measure (Dowdy et al., 2022), which found support for invariance across students based on sex, grade level, and Latinx status, supporting its use across diverse groups in schools.

Once gender and longitudinal invariance were established, this study provided evidence for gender differences in the SEDS-S. The gender differences found in this study were consistent with previous research showing that women are more likely to express internalizing symptoms, and men are more likely to express externalizing symptoms (Rocchino et al., 2017). Furthermore, the small effect sizes found for these gender differences are consistent with previous studies reporting the small magnitude of gender differences in adolescent internalizing problems (Piqueras et al., 2021a﻿). These findings suggest that gender differences, albeit small, should be considered when interpreting the SEDS-S results. For this reason, our study provides regulatory values for interpretation, differentiated by gender and age.

Regarding test–retest reliability, our study showed Cronbach’s and McDonald’s ω values between 0.86 and 0.89, which are slightly lower than those found in previous studies, but those studies only reported for the total sample, without differentiating according to gender (Chan et al., 2022a, 2022b; Dowdy et al., 2018; Furlong et al., 2020, 2021; Kim et al., 2019; Maupin, 2021). These data are consistent with those of the SEDS-S-Brief (Dowdy et al., 2022), which supported reliability, although based on temporal stability.

As regards the convergent and discriminant validity of the SEDS-S, the results show that this scale correlates positively with the distress measure and negatively with well-being. This finding is consistent with previous studies reporting that the SEDS correlates significantly with instruments assessing anxious and depressive symptomatology and negatively with measures of emotional well-being, life satisfaction, socio-emotional skills, and HRQoL (Chan et al., 2022a, 2022b; Dowdy et al., 2018; Furlong et al., 2020, 2021; Maupin, 2021). These data are consistent with those of the SEDS-S-Brief, which provided validity evidence based on a relationship with measures of positive psychological states, such as well-being or life satisfaction (Dowdy et al., 2022).

Finally, to our knowledge, this is the second study to provide information on centile scores, as the first was that of Furlong, Dowdy et al. (2022). Our findings (lowest 50% = 10–16; middle 25% = 17–21; and highest 25% = 22–40) are pretty similar to those reported by Furlong et al. (2022) that reported that students with SEDS-S scores between 10 and 19 were indicative of the lowest levels of distress (lowest 50%); students with SEDS-S scores between 20 and 26 comprised about the next 25% of students and were placed in a middle range; and the remaining about 25% of students reported experiencing the highest levels of distress with scores on the SEDS-S between 27 and 40. These normative data may help to localize emotional distress among adolescents. Thus, SEDS is a quick form of screening and has standardized values for its interpretation.

### Application

A key question that needs to be addressed here is how the SEDS-S as screening measure of symptom or distress factor can be used in a BMHM or DFM manner. This is, how the information provided by this article would be transferred to school mental health professionals about how they might use the SEDS in a BMHM/DFM manner. The answer to this question is that the SEDS-S asks students to rate their internal psychological experiences related to sadness (e.g., in the past month, I felt sad and down) and their anxious emotional experiences (e.g., I was scared for no good reason) (see Table 3). Consistent with the principles of screening efficiency, the SEDS-S assesses overall emotional distress to prioritize and identify students to allow for follow-up assessments and provide support services. An example of how the SEDS-S can be implemented in a BMHM/DFM manner is the proposal by Furlong et al. (2022) that suggests a 3 × 3 Dual-Factor Model for Universal Screening, where cut-off points were selected for the distress measure, the SEDS-S, and a measure of well-being, the Brief Multidimensional Students' Life Satisfaction Scale (BMSLSS, Huebner et al., 2004), namely a 25–25-50 cut-off point approach that allowed categorizing students into 3 categories for a 3 × 3 DFM: high (top 50%), medium (middle 25%) and low (lowest 25%). In this study, Furlong et al. (2022) reported that students with SEDS-S scores between 0 and 9 were indicative of the lowest levels of distress (lowest 50%); students with SEDS-S scores between 10 and 16 comprised about the next 25% of students and were placed in a middle range; and the remaining about 25% of students reported experiencing the highest levels of distress with scores on the SEDS-S between 17 and 30. The same logic was applied to create the 25–25-50 cut-score approach for the measure of wellness factor. According to the authors, this approach matches closely to past research providing additional information about youth in the middle ranges on indicators of distress and wellbeing.

### Limitations

A limitation of the present study is that the sample was limited to students from south-eastern Spain. On the other hand, only two waves of assessment were conducted in a short period (7 months). Hence, we recommend future studies to replicate the findings of longitudinal invariance over longer intervals.

## Conclusions

In conclusion, this study showed that the SEDS-S is a useful, brief, reliable, and valid screening measure for assessing emotional distress in adolescents. Specifically, this study provided evidence of the reliability and validity (structural and convergent-discriminant) of the Spanish version of the SEDS-S for adolescents. Furthermore, being a short measure, the SEDS-S can help assess distress in longitudinal research that requires instruments that do not tire the participants. It is, therefore, useful at the applied and research levels.

The incremental contribution of this work is the establishment of the psychometric properties of the Social Emotional Distress Scale-Secondary (SEDS-S) in a Spanish-speaking adolescent population. By providing evidence of its reliability, factor structure, convergent and discriminant validity, longitudinal and gender measurement invariance, and normative data, this study adds to the existing literature on mental health screening measures in Spanish-speaking populations. Thus, this work expands the applicability and utility of the SEDS-S beyond English-speaking populations, making it a valuable tool for mental health screening and program evaluation in diverse contexts beyond the school setting. Additionally, this study provides normative data that can serve as a reference point for future research and clinical practice in Spanish-speaking populations.

Overall, this study makes a significant contribution to the field of mental health assessment and has the potential to improve the accuracy and effectiveness of mental health screening and program evaluation in Spanish-speaking adolescent populations.

## Data Availability

For queries regarding data, contact the ‘author for correspondence’.
